# Serum deprivation/starvation leads to reactivation of HIV-1 in latently infected monocytes via activating ERK/JNK pathway

**DOI:** 10.1038/s41598-018-32316-2

**Published:** 2018-09-27

**Authors:** Rameez Raja, Sneh Lata, Shubhendu Trivedi, Akhil C. Banerjea

**Affiliations:** 1 Laboratory of Virology, National Institute of Immunology, New Delhi, India; 2 Present Address: Lerner Research Institute, Cleveland Clinic, Ohio, USA

## Abstract

Despite the high success rate, antiretroviral therapy does not cure the disease completely due to presence of latent viral reservoirs. Although several studies have addressed this issue earlier, the role of serum starvation/deprivation in HIV-1 latency has not been studied. So, we investigated the role of serum starvation in regulating HIV-1 latency. The impact of serum starvation on HIV-1 latency was assessed in latently infected monocytes U1 and T-cells J1.1. Serum starvation breaks HIV-1 latency in U1 cells. Under similar conditions, J1.1 cells failed to show reactivation of virus. We investigated the involvement of cell death pathway and autophagy during the serum starvation in viral reactivation. Inhibition of these pathways did not affect viral reactivation. Furthermore, other crucial factors like NF-κB, SP1 and AKT did not play any role in regulating viral latency. Here, we report that serum deprivation up-regulates ERK/JNK pathway. This leads to phosphorylation of c-Jun which plays an important role in viral reactivation. Treatment of cells with U0126, an ERK kinase inhibitor, potently inhibited viral replication. In summary, we show that serum starvation leads to reactivation of HIV-1 in latently infected monocytes through the ERK/JNK pathway.

## Introduction

Antiretroviral therapies have been able to prevent deaths in HIV-1 infected individuals but they are unable to cure it completely as withdrawal of drugs leads to rebound of the latent HIV-1^1^. HIV-1 latent reservoirs are mainly confined to CD4+ T cells and cells of monocyte-macrophage lineage^2,3^. Although, the HIV-1 reservoirs are small, approximately 1 in 1 × 10^6^ cells, they are sufficient to spread infection and cause disease when activated^3–5^. In the latent stage, viral replication may be suppressed at the pre-integration state by the host factors like APOBEC3G and SAMHD1 while at post-integration level, the viral latency is maintained by epigenetic changes such as DNA methylation, chromatin modeling etc.^4–6^. Strategies like activate and kill are being used to target the latently infected cells^6^. Cellular stresses like hyperthermia, amino acid starvation, DNA damage and apoptosis induction are known to promote viral replication or break the latency^7–9^. Hyperthermia inhibits replication in Vesicular Stomatitis Virus and Mayaro Virus while it is known to promote the replication of Rotavirus, Dengue virus, Epstein-Barr virus and Human Cytomegalovirus^10,11^. In HIV-1, heat shock activates viral transcription through Hsp90 which co-localizes with actively transcribing provirus and promotes viral replication^7^. Amino acid starvation has earlier been shown to regulate viral replication by affecting the process of acetylation. In the absence of amino acids, HDAC4, a de-acetylase, is down-regulated which relieves its inhibitory effect on silenced genes including HIV-1 proviral DNA. This effect is reported to be dependent on HDAC4 activity and only T-cells show reactivation while monocytic cell line U1 remains unaffected^8^. Induction of apoptosis also results in reactivation of latent HIV-1. This process is dependent on caspase-3 and caspase-8 and use of Z-VAD-FMK, a pan caspase inhibitor, was associated with decrease in HIV-1 replication^9^.

The role of growth factors in reactivation of HIV-1 latent pool is largely unknown. In case of Herpes Simplex Virus, neuronal growth factor deprivation leads to viral reactivation from the infected cells. This phenomenon is dependent on JNK (c-Jun N-terminal kinase) pathway, which operates through a methyl/phospho-switch, where histone phosphorylation initiates viral replication^12–15^. Other important signaling component which is activated during such stress conditions is ERK (Extracellular Signal-Regulated Kinase) pathway^16^. ERK/JNK kinases are members of MAPK (Mitogen-Activated Protein Kinases) family and are activated in response to cellular stress and cytokines^17,18^. The ERK/JNK kinases later affect their downstream target molecules like AP-1 (Activator protein 1) and other transcription factors as well^19–22^. AP-1 is a transcription factor of HIV-1 LTR promoter and is dimeric in nature^23–26^. ERK and JNK are known to target c-Jun, which is a critical component of AP-1, thereby affecting HIV-1 replication^19^. In HIV-1, activation of MAPK pathway is also known to enhance its infectivity through Vif dependent and Vif independent mechanism. MAPK mediated activation can be cell line specific. One example is activation of Ras/Raf pathway in HIV-1 infected monocytes, which then participates in activation of NF-κB and hence HIV-1 replication. Other example includes, p38/HOG MAPK regulated activation of HIV-LTR in T cells^27^.

**Figure 1:**
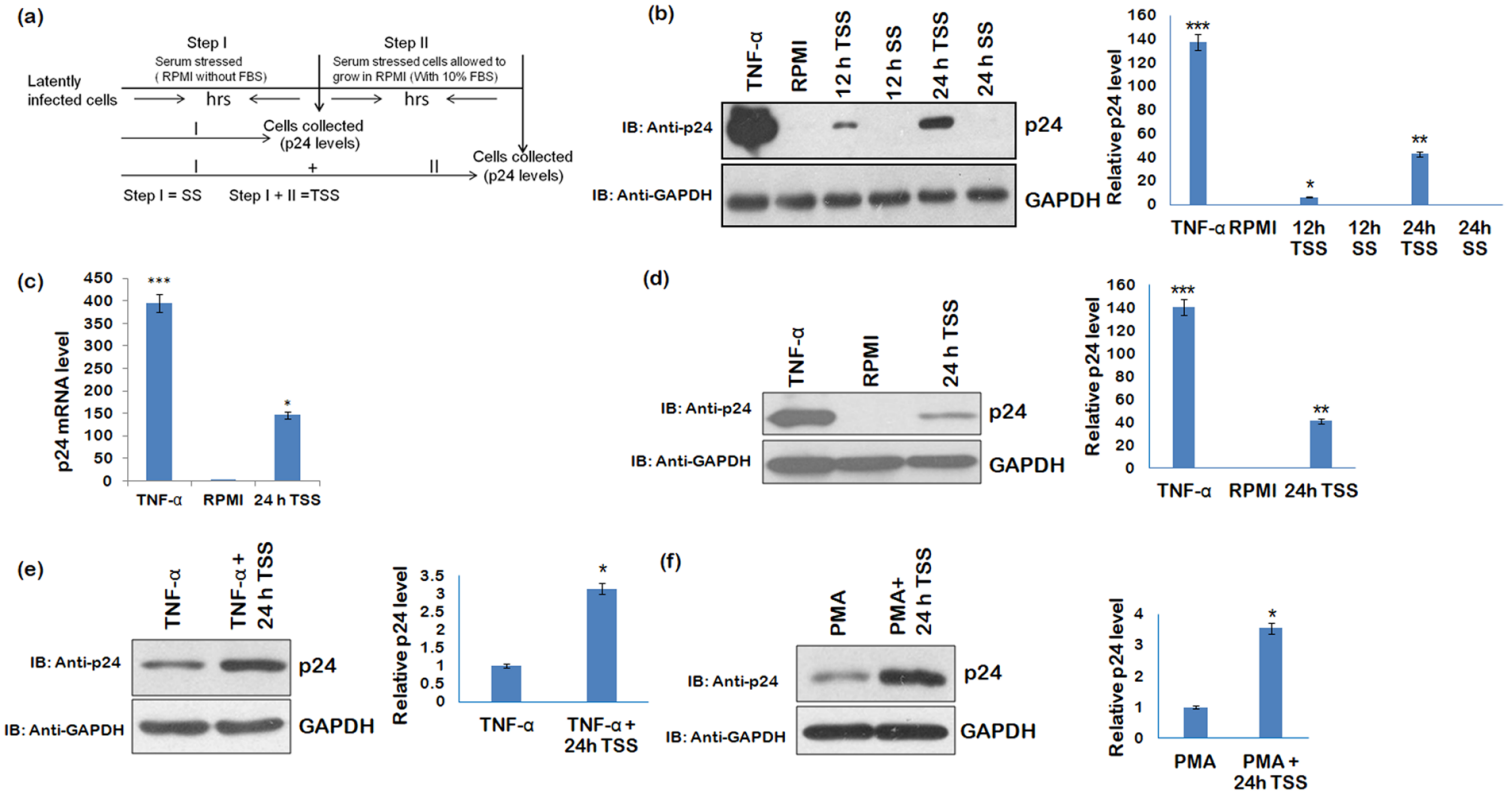
Transient serum stress reactivates HIV-1 in latently infected monocytic cell line U1. (**a**) Experimental design for the assessing viral reactivation. RPMI represents cells grown in RPMI + 10%FBS, SS represents RPMI without 10%FBS (Step I or complete serum starvation), while TSS represents serum stressed cells (SS) followed by growing these cells RPMI supplemented with 10%FBS (Step I + II or transient serum starvation) for next 24 h before harvesting them. (**b**) U1 (U937 derivative) cells were completely and transiently serum starved for 12 and 24 h. The cells were harvested and immunoblotted for p24 levels. GAPDH was used as a loading control. Right panel of Fig. 1b shows the fold change (**c**,**d**) U1 cells were serum starved for 24 h and then allowed to grow in complete medium (RPMI + 10%FBS) for next 24 h. The cells were collected after a total time of 48 h and divided into two parts. One part was used for western blot analysis to assess p24 levels. The other part was processed for mRNA analysis by using real-time qPCR as described in methods. Mean value and standard deviation were calculated from 2^dCt^ of three independent experiments. Right panel of Fig. 1d shows the fold change (**e**) U1 cells were treated with TNF-α alone (20 ng) or TNF-α along with transient serum stress (TSS). The cells were collected and analysed for p24 levels. Right panel of Fig. 1e shows the fold change (**f**) U1 cells were grown in RPMI + 10%FBS or transiently serum starved for 24 h and then treated with 50 ng PMA. The cells were harvested and p24 levels were assessed. GAPDH was used as a loading control. Right panel of Fig. 1d shows the fold change. p-values were calculated by a two-tailed t-test (*p < 0.05, **p < 0.01, ***p < 0.001). Full blots are shown in Supplementary Fig. 5.

The role of neuronal growth factors was earlier shown to be important in herpes simplex virus activation^12,13^. However, the effect of serum which is the main source of a number of important components like growth factors, hormones, amino acids etc. has not been tested before. We hypothesized that HIV-1 latency may also be controlled by serum components. We tested the effect of complete and temporary serum stress on HIV-1 reactivation in both the T-cell and monocytic cell line models (J1.1 and U1 respectively). The completely serum stressed cells (SS) and transiently serum stressed cells (TSS) (as illustrated in Fig. 1a) were used to investigate HIV-1 reactivation. Interestingly, serum deprivation was able to break HIV-1 latency in monocytic cell line U1 but not in T-cell line J1.1. We also investigated the various signaling pathways associated with reactivation of the latent virus.

## Materials and Methods

### Cell culture

U1 (a pro-monocytic cell line) and J1.1 (a T cell line) cells were cultured in RPMI (Himedia Laboratories) with or without 10% fetal bovine serum (Gibco, Invitrogen), 100 units penicillin, 0.1 mg streptomycin and 0.25 μg amphotericin B per ml at 37 °C in presence of 5% CO_2_ in a humidified incubator. Fetal Bovine Serum used during the experiments was not heat inactivated. This was done to avoid degradation of serum components. All experiments were performed on cell lines only.

### Western blot analysis

Western blot analysis was performed as described earlier^28,29^. The primary antibodies used were anti-AKT, anti-S473 phopsho-AKT, anti-GAPDH, anti-PARP, anti-Caspase-3, anti-ERK1/2, anti-phospho 44/42 ERK1/2, anti-phospho-SAPK/JNK, anti-SAPK/JNK, anti-SP1, anti-LC3B (Cell Signalling Technology), anti-p24 (Cat No. 6457, NIH), anti-IKBα (Santa Cruz Biotechnology) The secondary antibodies used were anti-rabbit/mouse-HRP conjugated (Jackson Immuno Research).

### Real-Time Polymerase Chain Reaction (RT-PCR)

Total RNA was isolated using Trizol reagent as described by the manufacturer’s protocol (Invitrogen) and reverse transcribed to form complementary DNA (cDNA) as described earlier^28^. This cDNA was used for real-time qPCR amplification using p24 specific primers: Forward Primer: 5′CAAGCAGCCATGCAAATGTT 3′, Reverse Primer: 5′ TGC TAT GTC ACT TCC CCT TGG 3′. Human β-actin was used as internal control and amplified using the following primers: Forward primer: 5′AGGCACCAGGGCGTGAT 3′, Reverse primer: 5′GCCCACATAGGAATCCTTCTGAC 3′.

### Chemicals

Human TNF-α (Sigma), PMA (Sigma), 3-Methyladenine (Sigma), U0126 (Cell Signalling Technology), Actinomycin D (Sigma), Doxorubicin (Sigma), Z-VAD-FMK (Calbiochem).

### FACS analysis

Flourimetric analysis was performed to assess the cell death using SYTOX Red dye. The cells were grown in the presence or absence of serum for 24 h and then stained with SYTOX Red dye for 15 min followed by FACS analysis. The dye was used at concentration of 5 μM.

### Serum starvation

The cells were grown in RPMI without FBS (SS) for different time points as illustrated in experimental design (Fig. 1). RPMI alone in figures represents RPMI + 10% FBS. Temporary/transient serum stress (TSS) represents serum stress followed by growing cells in medium supplemented with 10% FBS (RPMI +10% FBS).

### Statistical analysis

Results obtained were represented as mean ± SEM. p-value was calculated by a two-tailed *t*-test. Only values with p < 0.05 were considered significant.

## Results

### Serum deprivation reactivates HIV-1 replication in latently infected monocytes

U1 cells were serum starved for the indicated time periods and then analysed by western blot analysis for viral reactivation as shown (Fig. 1a). When the cells were cultured in media without serum (Step I, as explained in Fig. 1a), viral replication was not observed. Interestingly, viral reactivation was observed when the serum stressed cells were subsequently grown in the RPMI with 10%FBS for 24 h (Step I + II, as explained in Fig. 1a,b). Furthermore, U1 cells were also serum starved for longer time periods (36 h and 48 h) and allowed to grow in RPMI + 10%FBS for next 24 h as described earlier. The p24 levels were decreased when cells were subjected to serum stress for longer durations as shown in Supplementary Fig. 1a. When U1 cells were serum starved for shorter time durations, viral reactivation was observed (Supplementary Fig. 1b). Since serum stress for 24 h showed maximum effect on viral reactivation, we selected this time point for further experiments. We also tested the effect of serum stress on J1.1, a T-cell line with chronic HIV-1 infection as shown in Supplementary Fig. 2. No effect of serum deprivation was observed on the constitutive expression of HIV-1 in these cells as evident by almost similar levels of p24 in control and 24 h serum stressed cells. Thus, this phenomenon seems to be specific to monocytes only. Also, J1.1 cells are known to express detectable amounts of HIV-1 constitutively and therefore not considered ideal for studying HIV-1 latency^30^.

The effect of transient serum starvation on viral mRNA levels was also assessed by real-time qPCR. The cells were divided into two parts. Half of the cells were used for real-time qPCR analysis and remaining cells were used for western blot analysis. In J1.1 cells, there was no change in mRNA level of HIV-1 p24 upon serum stress (Supplementary Fig. 3) while p24 mRNA levels were significantly up-regulated in 24 h in U1 cells (Fig. 1c,d). This supports our earlier results that serum starvation can break latency in monocytic cells U1 but not in T-cells J1.1. We extended our study with the known reactivators of HIV-1 latency, namely TNF-α (Tumour necrosis factor-α) and PMA (phorbol 12-myristate 13-acetate)^31,32^. The U1 cells were treated with TNF-α alone or TNF-α along with transient serum stress (TSS). Serum stress enhanced the TNF-α mediated effect (Fig. 1e). Similarly, serum stress also enhanced PMA induced viral reactivation (Fig. 1f).

### Serum stress mediated reactivation of HIV-1 is independent of cell death and autophagy

**Figure 2:**
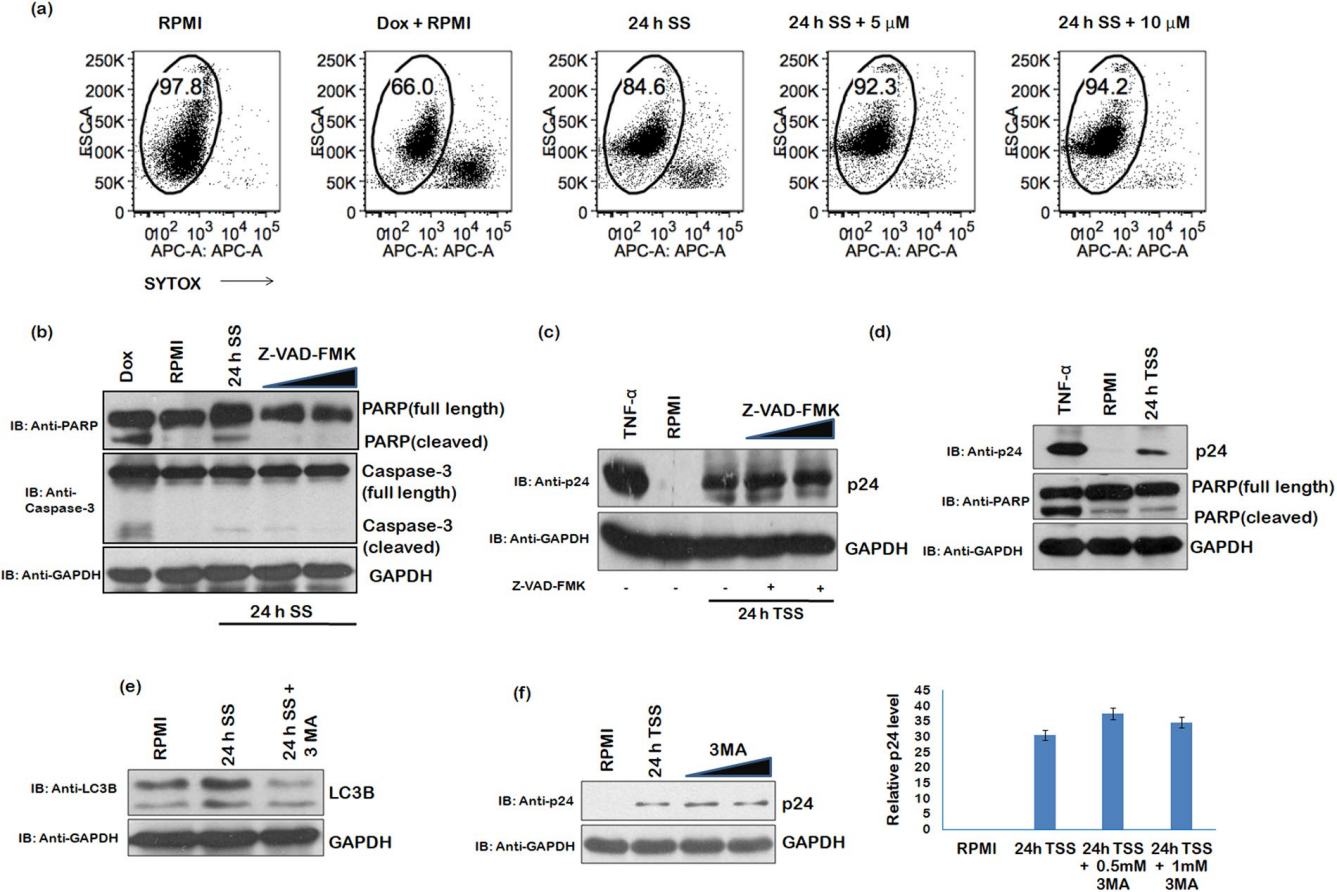
HIV-1 reactivation during serum stress is independent of cell death and autophagy. (**a**) 5 μM SYTOX Red dye was added to (**I**) U1 cells grown in RPMI (**II**) U1 cells grown in RPMI + doxorubicin treated (positive control) (**III**) U1 cells serum starved for 24 h (**IV**) U1 cells serum starved + Z-VAD-FMK (5 μM) (**V**) U1 cells serum starved + Z-VAD-FMK (10 μM) treated. FACS analysis was performed to assess the cell death. Gated population represents the percentage of live cells. (**b**) In parallel, half of the cells as shown in Figure (**a**) were processed by western blot analysis. The cells were collected and probed for PARP and Caspase-3 as a measure of apoptosis. (**c**) U1 cells were cultured in RPMI (10%FBS), U1 cells were treated with Z-VAD-FMK (5 μM and 10μΜ) during serum starvation to inhibit cell death and then allowed to grow in RPMI (with 10%FBS). TNF-α was used as a positive control. The cells were harvested and probed by western blot analysis for p24 levels. (**d**) U1 cells were serum starved for 24 h and then cultured in RPMI + 10%FBS for next 24 h (TSS). The cells were lysed and probed for PARP levels by western blot analysis. (**e**) After 24 h of serum starvation, U1 cells were lysed and probed for LC3B levels to assess the extent of autophagy. 3-MA was used at 0.5 mM conc. to inhibit autophagy. (**f**) U1 cells were serum starved for 24 h in presence of 0.5 mM and 1 mM 3-MA to inhibit autophagy activated in these conditions. The cells were then allowed to grow in RPMI supplemented with 10%FBS. The cells were collected 24 h post serum starvation and subject to western blot analysis to assess p24 levels. GAPDH was used as a loading control. Right panel of Fig. 2f shows the fold change. Full blots are shown in Supplementary Fig. 6.

To assess whether cell death is associated with viral reactivation, if any, under serum stress conditions, FACS analysis was performed using SYTOX Red dye. Half of the U1 cells were used for FACS analysis and the other half was subjected to western blot analysis. FACS analysis showed approximately 13% cell death in 24 h serum starved U1 cells compared to control cells grown in RPMI (with 10%FBS) (Fig. 2a, panel I and III). Doxorubicin was used to induce cell death (Fig. 2a, panel II). Z-VAD-FMK (a pan-caspase inhibitor) was used to inhibit the cell death as shown in panel IV and V (Fig. 2a). The levels of caspase-3 and PARP protein were probed to assess the cell death by western blot analysis and to confirm effect of Z-VAD-FMK on caspase-3 and PARP (Fig. 2b). Further, to rule out the involvement of cell death pathways in viral reactivation, we incubated U1 cells with Z-VAD-FMK during serum stress conditions. After growing these cells in RPMI (with 10%FBS) for next 24 h, the cell lysates were analyzed by western blotting for p24 levels. Z-VAD-FMK did not affect viral reactivation (Fig. 2c), thus confirming that serum stress reactivates HIV-1 independent of cell death. PARP levels in transiently serum starved cells (TSS) were also unaffected (Fig. 2d).

Since, serum stress also induces autophagy^33^, so we investigated the role of autophagy in serum stress mediated viral reactivation. As expected, LC3B, an indicator of autophagy, was increased after serum starvation^34^. 3-Methyladenine (3-MA) was used to suppress serum starvation induced autophagy (Fig. 2e)^35^. After treatment of U1 cells with 3-MA during serum starvation, p24 levels were not affected (Fig. 2f). These results indicated that autophagy did not play any role in latent HIV-1 reactivation.

### Role of Signalling Molecules/Transcription factors in HIV-1 reactivation

**Figure 3:**
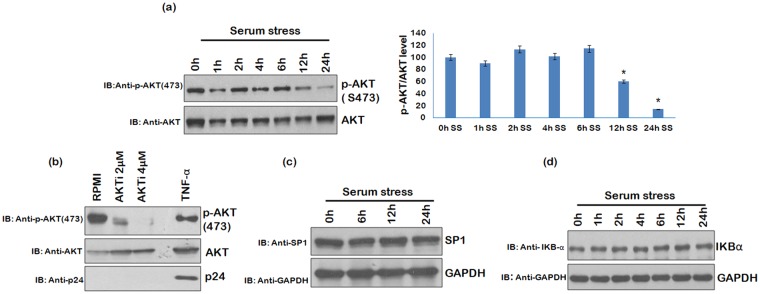
Involvement of transcription factors and signalling molecules in HIV-1 reactivation. (**a**) U1 cells were serum starved for different time periods and then probed for p-AKT (S473) levels by western blot analysis. Total AKT was used as a loading control (*p < 0.05) (**b**) U1 cells grown in RPMI +10%FBS were treated with 2 μM and 4 μM AKTi for 24 h and probed for p24 levels. Total AKT was used as a loading control. Right panel of Fig. 3a shows the fold change. (**c**) SP1 levels were assessed at different time points in serum starved cells by western blot analysis using anti-SP1 antibody. GAPDH was used as a loading control. (**d**) U1 cells were serum starved for indicated time points and probed for the IKB-α level by western blot analysis. GAPDH was used as a loading control. Full blots are shown in Supplementary Fig. 7.

The experiments involving pan caspase inhibitor Z-VAD-FMK and autophagy inhibitor clearly established that the viral reactivation is independent of cell death and autophagy. Next, we investigated the role of certain key transcription factors or other signalling molecules in viral reactivation in serum stressed cells. The levels of AKT, SP1 and NF-κB were analyzed at different time points in serum stressed U1 cells as they were earlier shown to be important in HIV-1 reactivation^32,36–38^. Phosphorylation of AKT was down-regulated in 24 h serum starved cells (Fig. 3a). To find out its importance for viral reactivation, U1 cells grown in RPMI (with 10%FBS) were treated with AKT inhibitor (AKTi) for 24 h without serum stress. AKTi treatment of U1 cells had no effect on viral reactivation as shown in Fig. 3b, thus, excluding the role of AKT in latent viral reactivation in our study. Another transcription factor SP1 showed no change during the serum starvation (Fig. 3c). To investigate the role of NF-κB signalling, we assessed IKBα levels which were also unchanged during serum stress conditions (Fig. 3d). Since SP1 and NF-κB levels remain unaffected during serum stress, we conclude that they have no role in viral reactivation in U1 cells.

### Phosphorylation of ERK/JNK is up-regulated during serum starvation which correlates viral reactivation

**Figure 4:**
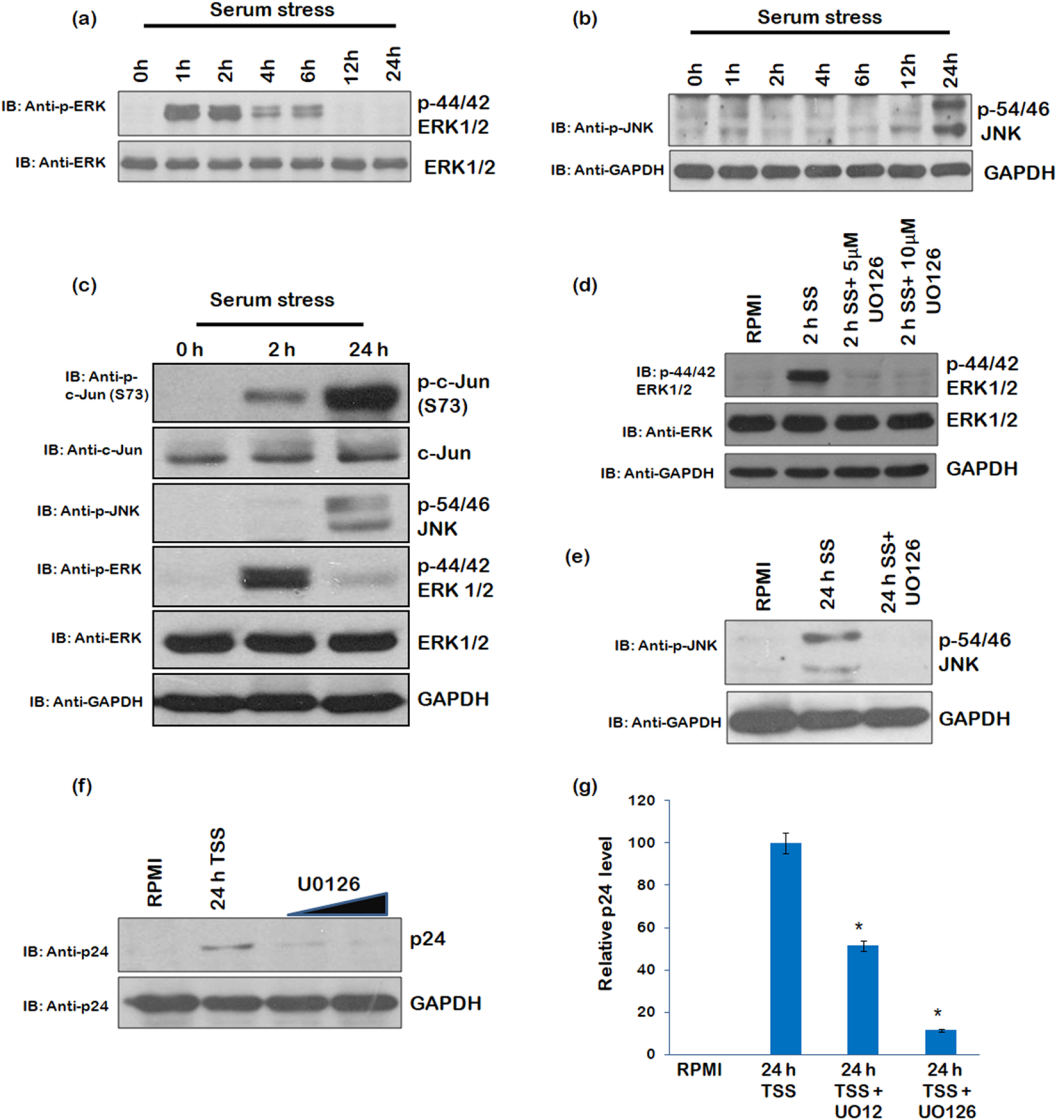
ERK/JNK phosphorylation is up-regulated during serum starvation and correlates with HIV-1 reactivation. (**a**) U1 cells were completely serum starved for indicated time points and probed for phospho-ERK (44/42) levels by performing western blot analysis. Total ERK was used as a loading control. (**b**) p-JNK (54/46) levels were assessed at different time points in serum starved U1 cells by western blot analysis. (**c**) U1 cells were serum starved for 0, 2 and 24 h and then probed with the indicated antibodies. (**d**) U1 cells were serum starved for 2 h and treated with 5 μM and 10 μM U0126 to inhibit ERK phosphorylation. The cells were collected and lysed. Western blot analysis was performed to probe phospho-ERK (44/42). Total ERK was used as loading control. (**e**) U1 cells were serum starved for 24 h and treated with 5 μM U0126 during the serum starvation to inhibit JNK phosphorylation. The cells were harvested and analysed for phospho-JNK (54/46) by performing western blot analysis. (**f**) U1 cells were treated with 5 μM and 10 μM U0126 during the 24 h of serum starvation. After allowing the U1 cells to grow in RPMI + 10%FBS for a period of 24 h, the cells were collected and immunoblotted with anti-p24 antibody. GAPDH was used as a loading control. (**g**) Densitometry of the western blots of Figure (**f**) using Image J software (*p < 0.05, **p < 0.01). Full blots are shown in Supplementary Fig. 8.

Since growth factor deprivation can activate ERK/JNK signalling pathway^16^, so we investigated the role of ERK/JNK signalling in latent HIV-1 reactivation under serum stress conditions. In the initial phase of serum starvation, phosphorylation of ERK1/2 (44/42) increased while it decreased during the later phase (Fig. 4a). Phospho-JNK (54/46) levels were also analyzed at different time points of serum starvation. Initially there was no change in JNK phosphorylation but a significant increase in phospho-JNK (54/46) was observed during the later stages (Fig. 4b). In addition, we also analyzed the extent of c-Jun phosphorylation during serum starvation conditions since it is a common target of ERK and JNK^19^. c-Jun is one of the subunits of transcriptional factor AP-1^26^. We observed that serum starvation increased c-Jun phosphorylation at both the early and later time points as shown in Fig. 4c. This phosphorylation is known to enhance AP-1 transcriptional activity^39^. In case of J.1.1 cells (T-cells), the increase in ERK/JNK and c-Jun phosphorylation was marginal (Supplementary Fig. 4). When serum starved U1 cells were treated with U0126 for different time periods to inhibit ERK/JNK activation^19,39^, both phospho-ERK (44/42) and phospho-JNK (54/46) levels were down-regulated as shown (Fig. 4d,e).

Since phospho-ERK (44/42) and phospho-JNK (54/46) levels were up-regulated in the serum starved U1 cells, we investigated the role of ERK and JNK phosphorylation in HIV-1 latency. To confirm the role of ERK/JNK signalling in latent HIV-1 reactivation, U1 cells were treated with U0126, an inhibitor of ERK/JNK activation, during serum starvation. This led to a decrease in the p24 levels (Fig. 4f,g). These results indicate that ERK/JNK signalling pathway is involved in latent HIV-1 reactivation under serum stress conditions.

### HIV-1 mRNA production is initiated in serum stressed conditions

**Figure 5:**
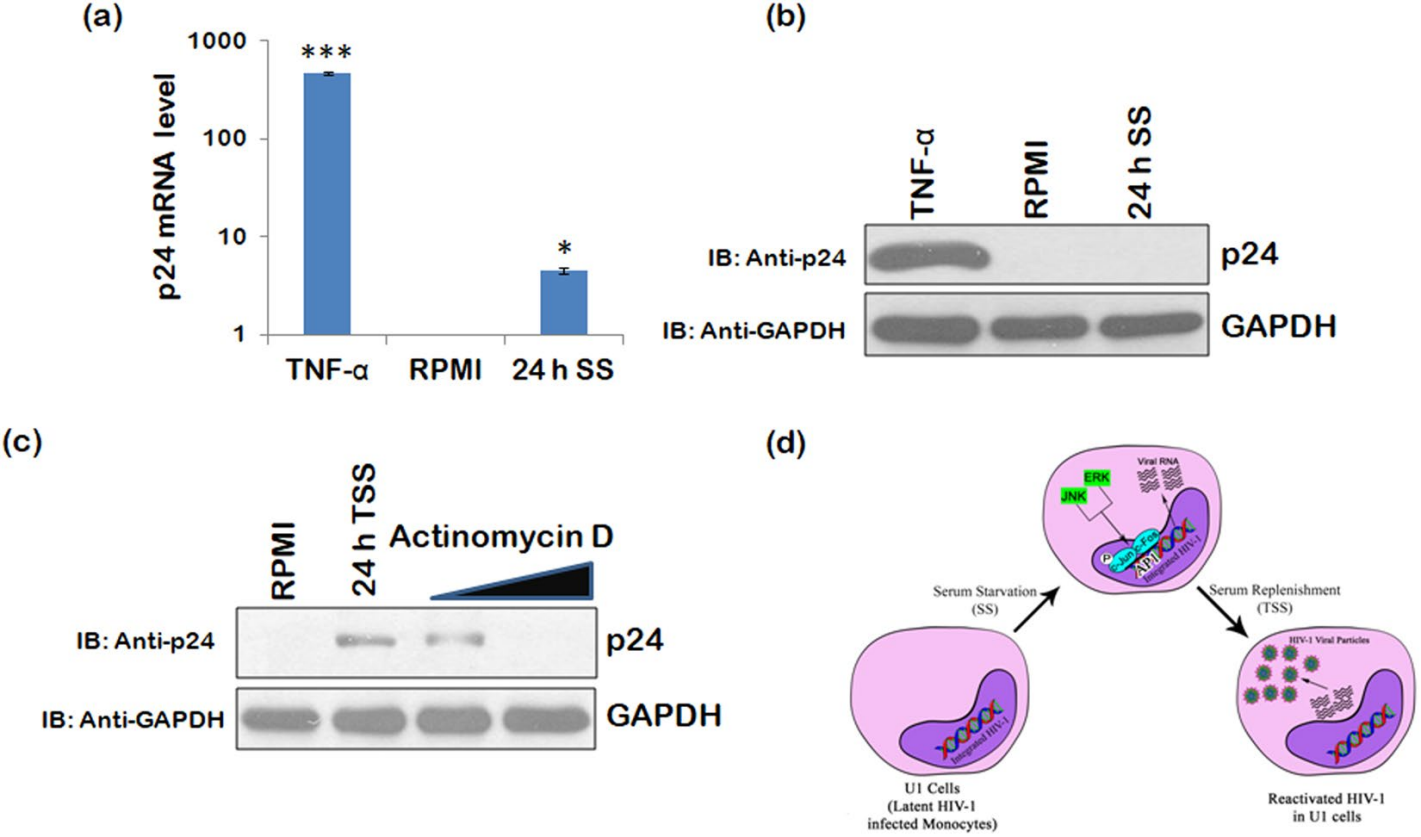
HIV-1 mRNA synthesis is induced during serum starvation. (**a**) U1 cells were serum starved for 24 h and lysed in Trizol reagent for mRNA extraction. Real-time qPCR was performed as described in methods. (**b**) Half of the cells were processed for western blot analysis using anti-p24 antibody. (**c**) U1 cells were treated with Actinomycin D (0.25 μg and 0.5 μg) during serum starvation and then allowed to grow in RPMI supplemented with 10%FBS for the next 24 h. The cells were harvested and subjected to western blot analysis using p24 antibody. GAPDH was used as a loading control. p-values were calculated by a two-tailed t-test (*p < 0.05, ***p < 0.001). (**d**) Mechanistic model showing serum stress induced HIV-1 reactivation in latently infected monocytes. HIV-1 latency is one of the major hurdles to treat AIDS. In this study, we discover a new mechanism which can initiate viral reactivation in latently infected monocytes. This approach is similar to shock and kill strategies used to target HIV-1 latent pool and appears to specifically target latently infected monocytes. Here, in this model we show that during serum deprivation/serum stress, phosphorylation of ERK and JNK is upregulated which then induce phosphorylation of c-Jun, thereby activating HIV-1 transcription factor AP1. Activation of AP1 initiates viral transcription. When the medium was replenished with serum, viral reactivation was observed as shown by p24 levels. Full blots are shown in Supplementary Fig. 9.

ERK/JNK signalling pathway was earlier found to be up-regulated during serum stress conditions which further increased phosphorylation of c-Jun (subunit of transcriptional factor AP-1), which suggests that ERK/JNK pathway might be influencing the viral reactivation through AP-1. So, we investigated mRNA levels of HIV-1 p24 under these conditions. The cells were divided into two parts. Half of cells were used for real-time qPCR analysis and the remaining cells were used for western blot analysis. In serum stressed cells, an increase in p24 mRNA levels was observed, but there was no change in the level of p24 protein expression (Fig. 5a,b). These results indicate that serum deprivation leads to the initiation of viral mRNA synthesis. To further validate this, cells were treated with Actinomycin D during the serum deprivation and then allowed to grow normally in RPMI + 10%FBS. Actinomycin D treatment was able to completely inhibit HIV-1 reactivation as shown in the Fig. 5c.

## Discussion

ART drugs can only target the actively transcribing virus but are unable to target the latent viral reservoir rendering HIV-1 cure very difficult^40^. The strategies like hyperthermia, amino acid starvation, apoptosis and growth factors deprivation have earlier been shown to reactivate the replication of some of the viruses^7–9^, but the role of serum was not investigated earlier. We tested the possibility of involvement of serum components in HIV-1 latency by creating serum stress conditions. Initially the cells were serum starved to induce nutrient stress and then these cells were allowed to grow in RPMI supplemented with 10%FBS as illustrated in the results section. By using this strategy, we found that U1 cells (human monocytic cell line latently infected with HIV-1) showed reactivation of the latent virus but in J1.1 cells (human T-cell line latently infected with HIV-1), viral replication was unaffected. Thus, this phenomenon was specific to monocytes only. In addition, other T-cell line models which do not show leaky expression can be more useful for studying HIV-1 reactivation.

Earlier studies have also reported cell line specific reactivation mechanisms in HIV-1. Amino acid starvation is reported to have differential effect on U1 and J1.1^8^. In that study it was shown that amino acid starvation reactivates HIV-1 in T cells but not in monocytes. This was attributed to the presence of HDAC4 (Histone deacetylase 4) in T-cells but not in monocytes. In our study, the duration of serum stress was also found to have differential effect on HIV-1 reactivation. Serum stress for shorter duration was able to break the viral latency but the effect was profound when the cells were serum starved for longer durations. Moreover, the transient serum stress showed synergistic effect along with known latency reactivators like TNF-α and PMA. HIV-1 p24 levels increased up to 3 fold upon TNF-α or PMA treatment in case of transiently serum starved cells when compared with U1 cells grown in serum enriched RPMI. We also investigated various cellular pathways and processes which are known to be affected during serum starvation. Since cells in nutrient deficient (serum starvation) conditions are prone to cell death, so we explored the involvement of cell death pathway in serum starved cells that exhibited HIV-1 reactivation. PARP and Caspase-3 protein levels under serum stress conditions along with the results of FACS analysis, using SYTOX red dye, revealed that serum stress caused cell death to some extent. To validate the role of cell death pathway and involvement of caspases, if any, we treated serum stressed cells with pan caspase inhibitor, Z-VAD-FMK. The treatment of cells with Z-VAD-FMK was able to inhibit cell death; however, it failed to suppress the reactivation of HIV-1. p24 levels remained unaffected in presence of pan caspase inhibitor. This finding suggests that cell death may not be playing any major role in serum stress induced HIV-1 reactivation in monocytes. Next, we assessed the role of autophagy in serum stress mediated HIV-1 reactivation. Serum stress is known to induce autophagy. Therefore, we probed for the levels of LC3B during the viral reactivation process. LC3B levels were increased during serum starvation indicating up-regulation of autophagy. The impact of autophagy on HIV-1 reactivation was monitored by using the autophagy inhibitor, 3-Methyl adenine (3-MA). To our surprise, inhibition of autophagy by 3-MA did not affect viral reactivation. From this result, we ruled out the role of autophagy.

While we continued our efforts to find out the mechanism of serum stress induced reactivation of HIV-1 in monocytes, further we decided to study the role of known positive regulators of viral replication. Role of NF-κB, AKT, and SP1 in viral reactivation was ruled out by subsequent experiments. NF- κB and SP1 levels remained unchanged during serum starvation while p-AKT levels decreased after prolonged serum starvation. When AKT inhibitor treatment was given to U1 cells grown in RPMI (10%FBS) without serum stress, we observed reduction in p-AKT levels with AKT inhibitor which was also the result of serum stress (Fig. 3a), however, viral reactivation was not seen (Fig. 3b). This ruled out the role of p-AKT in serum stress induced HIV-1 reactivation. Cells respond to stress by up-regulating certain survival pathways, for example, MAPK pathway plays important role in regulating cell survival. We investigated the role of ERK/JNK pathway in serum stress mediated HIV-1 reactivation as it is known to enhance AP-1 transcriptional activity through c-Jun phosphorylation. Interestingly, we found that p-ERK levels were up-regulated in the initial phase of serum starvation followed by up-regulation of the p-JNK in the later stage. Then, we investigated the involvement of ERK/JNK pathway in latency breakdown by using U0126^41^. Inhibition of the ERK/JNK pathway by U0126 led to suppression of the viral reactivation, indicating the role of this pathway in serum stress mediated viral reactivation. Phosphorylation of c-Jun (subunit of the transcriptional factor AP-1), which is a target of ERK/JNK signalling pathway, was also found to be up-regulated during serum stress. Since phosphorylation of c-Jun increases the transcriptional activity of AP-1, so we assessed the viral mRNA levels under stress conditions. Real-time qPCR analysis showed increase in viral mRNA even under serum stress conditions, however, change in protein levels was observed only when the serum stressed U1 cells were allowed to grow in media supplemented with 10% FBS. Furthermore, the change at mRNA level was more profound when the serum stressed cells were allowed to grow in RPMI supplemented with 10% FBS. Yang X *et al*. has also reported the role of MAPK signalling pathways to be important for HIV-1 pathogenesis. Activation of Ras/Raf pathway in HIV-1 infected monocytes promotes viral replication through NF-κB. On the other hand, p38/HOG MAPK signaling is needed for HIV-1 replication in T-cells^27^.

In summary, we show here that serum starvation leads to the reactivation of latent HIV-1 replication via up-regulation of ERK and JNK signalling in cells of monocytic lineage only and not T-cells. Also, we report a new strategy of serum deprivation that can be used to target the monocytes which constitute the latent reservoir of HIV-1 (Fig. 5d). Serum starvation/depletion can be used to reactivate the virus from the latent cells which can then be effectively controlled by antiretroviral treatment.

## Electronic supplementary material

Supplementary figures
