# Supplementary material for: Serum deprivation/starvation leads to reactivation of HIV-1 in latently infected monocytes via activating ERK/JNK pathway
Source: Sci Rep. 2018 Sep 27;8:14496. doi: 10.1038/s41598-018-32316-2 (PMC6160481; doi:10.1038/s41598-018-32316-2)
Supplement: Supplementary file 1 — Supplementary figures [file 41598_2018_32316_MOESM1_ESM.doc]

**Serum deprivation/starvation leads to reactivation of HIV-1 in latently infected monocytes via activating ERK/JNK pathway**

Rameez Raja^1#^, Sneh Lata^1^, Shubhendu Trivedi^1^, Akhil C. Banerjea^1*^

**Author affiliation:**

^1^Laboratory of Virology, National Institute of Immunology, New Delhi, India

#Current address: Lerner Research Institute, Cleveland Clinic, Ohio, USA

**^*^Corresponding author**

Akhil C. Banerjea, Laboratory of Virology, National Institute of Immunology, New Delhi-110067, India. Ph: 91-11-26703616; Fax: 26742125

Email ID: [akhil@nii.res.in](mailto:akhil@nii.res.in)

**
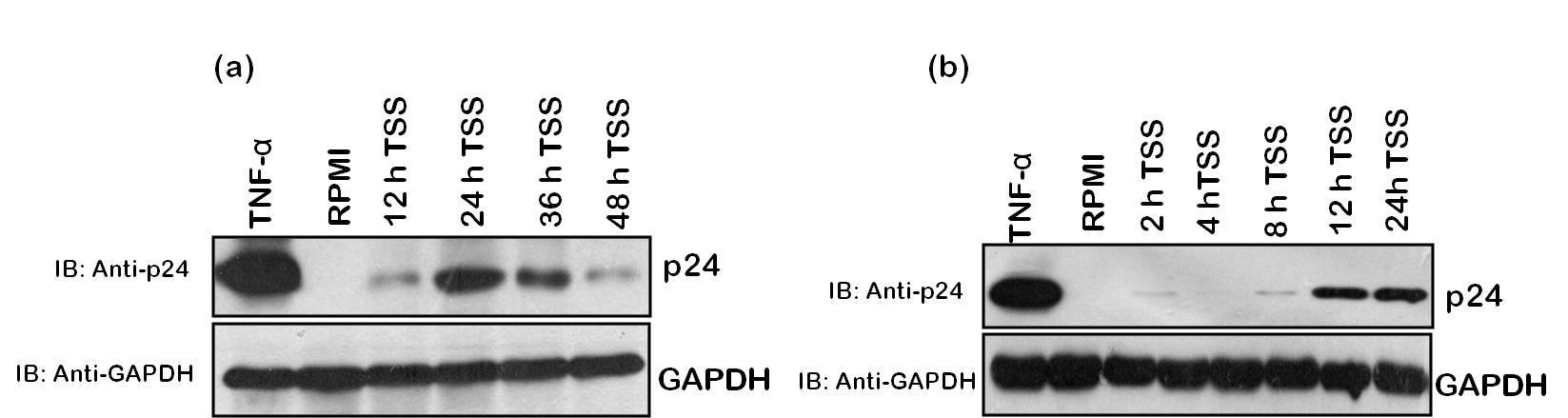
**

**Supplementary figure 1 Effect of serum starvation time on HIV-1 reactivation in U1. (a)** U1 cells were serum stressed for indicated time periods and then allowed to grow in RPMI supplemented with 10%FBS for next 24 h. Cells were harvested and subjected to western blot analysis. **(b)** U1 cells were serum starved for shorter time durations (2 to 24 h) and then allowed to grow in RPMI + 10%FBS for 24 h. The cells were harvested and immunoblotted with p24 antibody to assess viral reactivation. GAPDH was used as a loading control. Full blots are shown in supplementary figure 10.


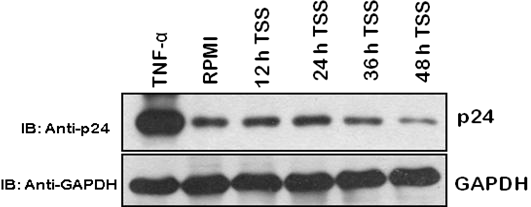


**Supplementary figure 2 Effect of serum starvation time on HIV-1 replication in J.1.1 (a)** J1.1 cells were serum starved for different time durations (12 to 48 h) and then allowed to grow in RPMI + 10%FBS for next 24 h. The cells were harvested and immunoblotted with p24 antibody to assess viral reactivation. GAPDH was used as a loading control. Full blots are shown in supplementary figure 11.


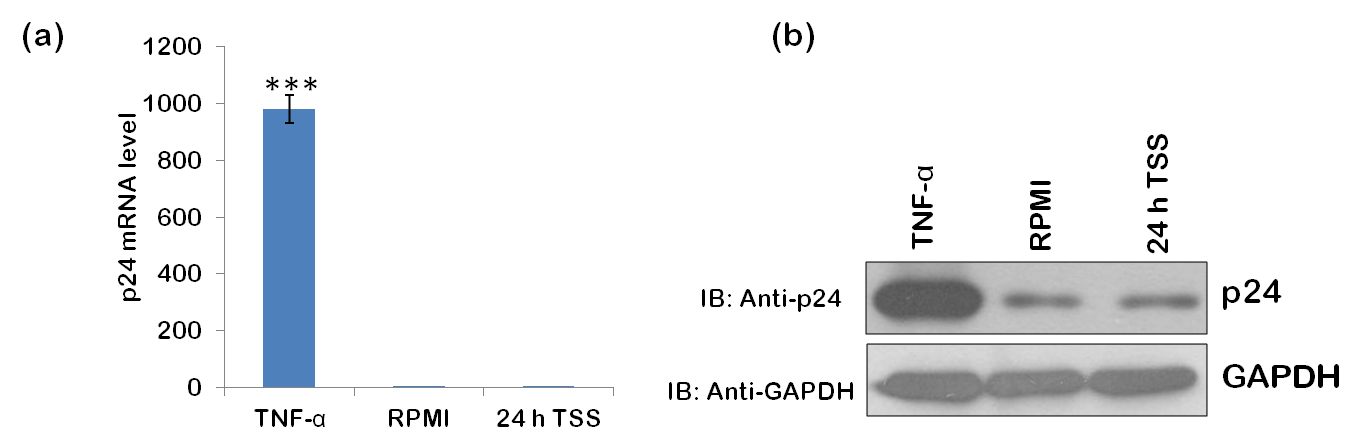


**Supplementary figure 3 Serum starvation does not affect HIV-1 reactivation in J.1. (a) (b)**

J.1.1 cells were serum starved for 24 h and then allowed to grow in complete medium (RPMI + 10%FBS) for next 24 h. The cells were collected after a total time period of 48 h and divided into two parts. One part was used for western blotting analysis to assess p24 levels. GAPDH was used as a loading control. The second part was processed for mRNA analysis by using real-time qPCR as described in methods. Mean value and standard deviation were calculated from 2^dCt^ of three independent experiments. p-values were calculated by a two-tailed t-test (*p<0.05,

***p<0.001). Full blots are shown in supplementary figure 12.


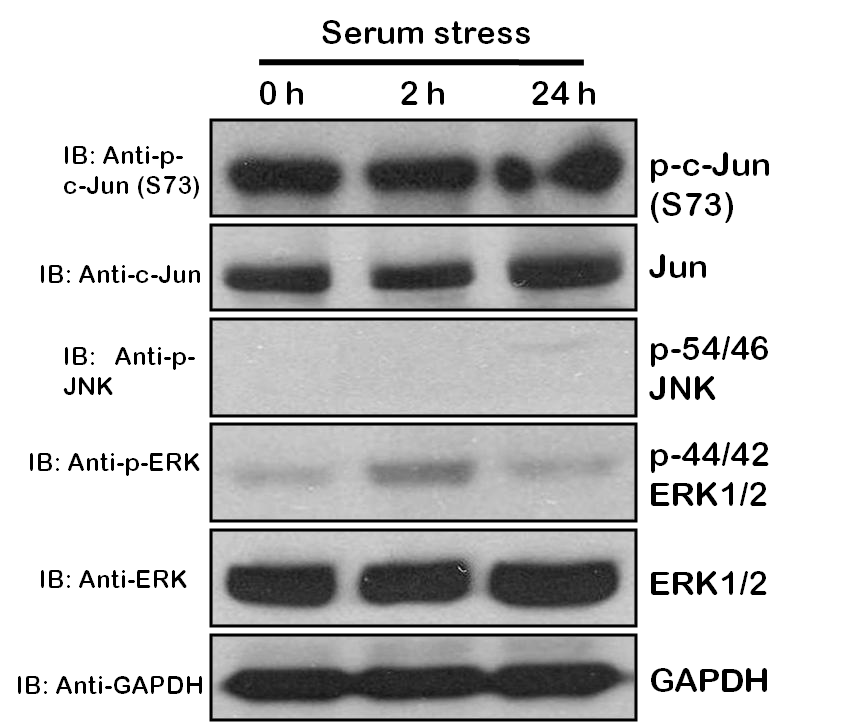


**Supplementary figure 4 ERK/JNK pathway is marginally up-regulated during serum starvation in J1.1 (a)** J1.1 cells were completely serum starved for indicated time points (0, 2

and 24 h) and probed for phospho-ERK (44/42) levels, p-JNK and p-c-Jun levels by performing western blot analysis. Total ERK, total Jun and GAPDH were used as a loading control. Full blots are shown in supplementary figure 13.


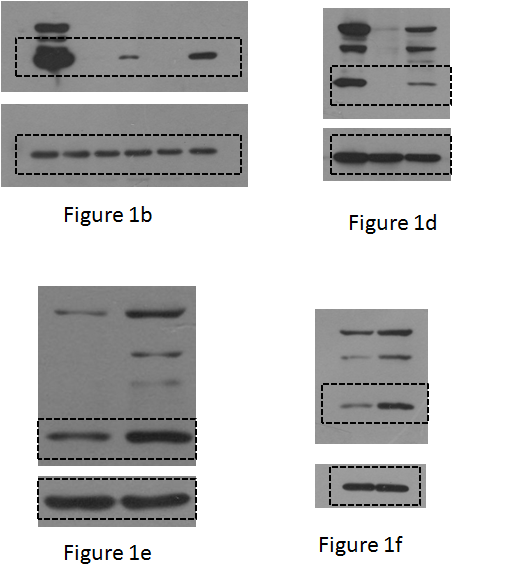


**Supplementary figure 5** Full blot images of results shown in Figure 1


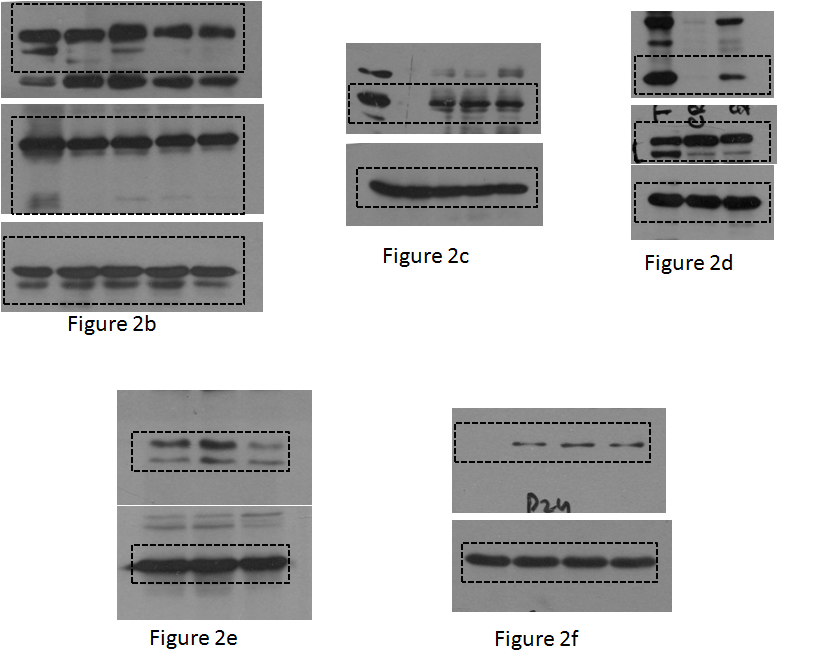


**Supplementary figure 6** Full blot images of results shown in Figure 2


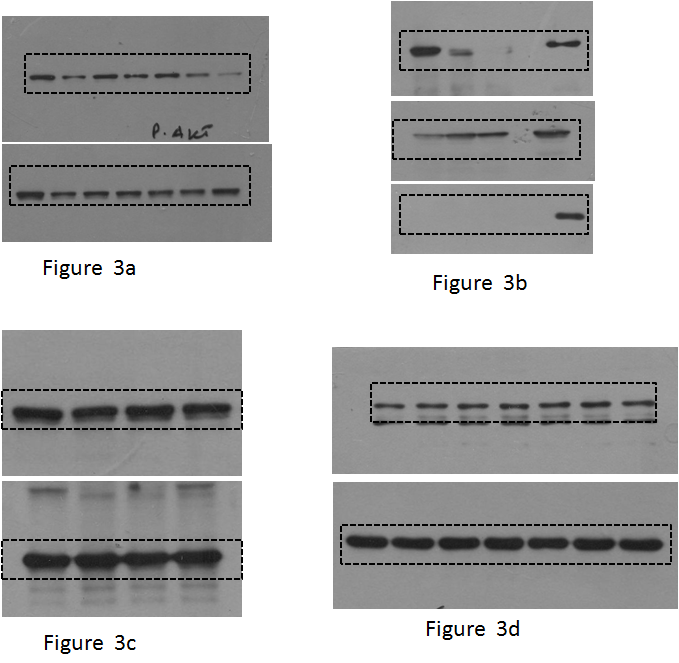


**Supplementary figure 7** Full blot images of results shown in Figure 3


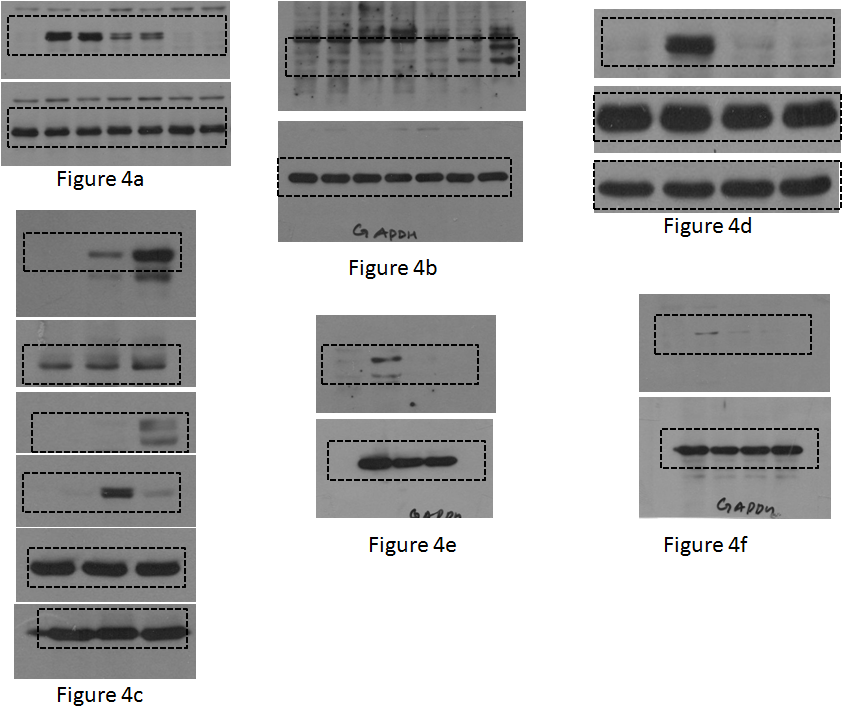


**Supplementary figure 8** Full blot images of results shown in Figure 4


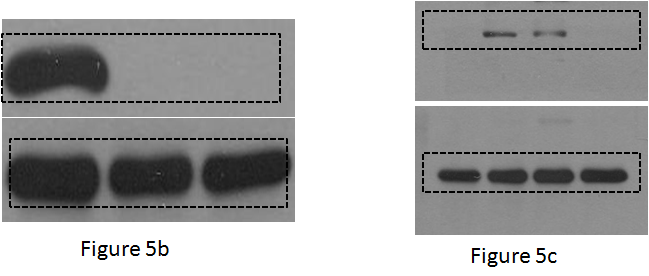


**Supplementary figure 9** Full blot images of results shown in Figure 5

**
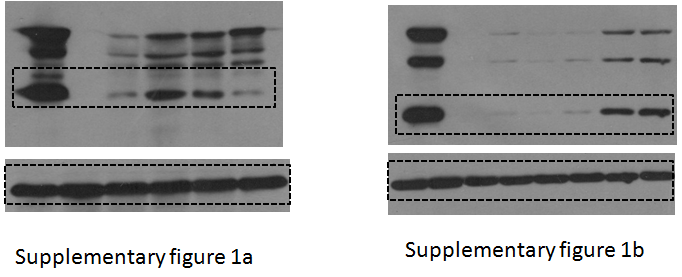
**

**Supplementary figure 10** Full blots images of results shown in supplementary figure 1


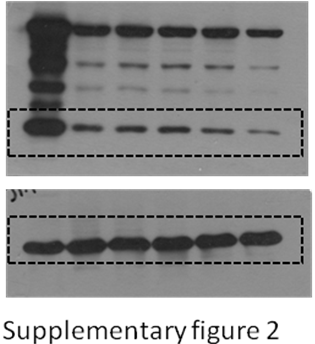


**Supplementary figure 11** Full blots images of results shown in supplementary figure 2


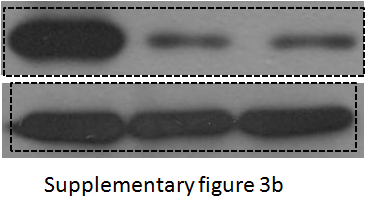


**Supplementary figure 12** Full blots images of results shown in supplementary figure 3

**
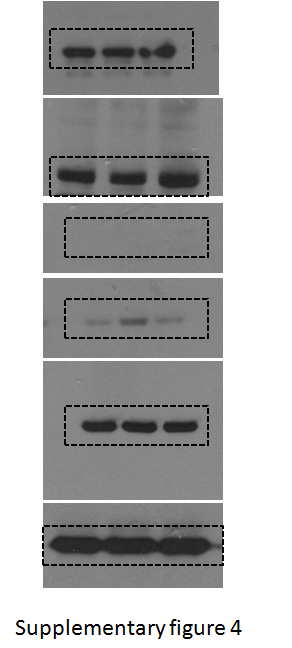
**

**Supplementary figure 13** Full blots images of results shown in supplementary figure 4
